# New Biosourced Flame Retardant Agents Based on Gallic and Ellagic Acids for Epoxy Resins

**DOI:** 10.3390/molecules24234305

**Published:** 2019-11-26

**Authors:** Valeriia Karaseva, Anne Bergeret, Clément Lacoste, Hélène Fulcrand, Laurent Ferry

**Affiliations:** 1 INRA, UMR 1083 SPO, 2 place Pierre Viala, 34060 Montpellier, France; 2 Centre des Matériaux des Mines d’Alès (C2MA), IMT Mines Alès, Université de Montpellier, 6 avenue de Clavières, 30319 Alès cedex, France; anne.bergeret@mines-ales.fr (A.B.); clement.lacoste@mines-ales.fr (C.L.); 3 French Environment and Energy Management Agency, 20 avenue du Grésillé, BP 90406, 49004 Angers cedex 01, France; 4 INRA, UMR 1208 IATE, 2 Place Pierre Viala, 34060 Montpellier, France; helene.fulcrand@inra.fr

**Keywords:** biobased flame retardant, ellagic acid, gallic acid, boration, epoxy resin

## Abstract

The aim of this work was an investigation of the ability of gallic (GA) and ellagic (EA) acids, which are phenolic compounds encountered in various plants, to act as flame retardants (FRs) for epoxy resins. In order to improve their fireproofing properties, GA and EA were treated with boric acid (to obtain gallic acid derivatives (GAD) and ellagic acid derivatives (EAD)) to introduce borate ester moieties. Thermogravimetric analysis (TGA) highlighted the good charring ability of GA and EA, which was enhanced by boration. The grafting of borate groups was also shown to increase the thermal stability of GA and EA that goes up respectively from 269 to 528 °C and from 496 to 628 °C. The phenolic-based components were then incorporated into an epoxy resin formulated from diglycidyl ether of bisphenol A (DGEBA) and isophorone diamine (IPDA) (72, 18, and 10 wt.% of DGEBA, IPDA, and GA or EA, respectively). According to differential scanning calorimetry (DSC), the glass transition temperature (T_g_) of the thermosets was decreased. Its values ranged from 137 up to 108 °C after adding the phenolic-based components. A cone calorimeter was used to evaluate the burning behavior of the formulated thermosets. A significant reduction of the peak of heat release rate (pHRR) for combustion was detected. Indeed, with 10 wt.% of GA and EA, pHRR was reduced by 12 and 44%, respectively, compared to that for neat epoxy resin. GAD and EAD also induced the decrease of pHRR values by 65 and 33%, respectively. In addition, a barrier effect was observed for the resin containing GAD. These results show the important influence of the biobased phenolic compounds and their boron derivatives on the fire behavior of a partially biobased epoxy resin.

## 1. Introduction

Due to their intrinsic characteristics, such as mechanical and electrical insulating properties, chemical resistance under both acidic and basic conditions, and adhesive properties, epoxy resins are widely used for structural applications (in association with glass or carbon fibers), as well as in the coating, electronic, and adhesive industries [1]. However, low thermal and fire resistance of epoxy resins are drawbacks limiting their use in some applications. To improve these properties, the addition of flame retardants (FRs) has been practiced for many years.

Among others, halogenated FRs have been extensively used [2,3]. But the harmful effects on both environment and human beings are an impediment to their use [4]. Thus, the development of halogen-free FRs, ideally extracted from renewable resources, is a subject of extensive investigation for epoxy resins [5,6,7].

**Figure 1:**
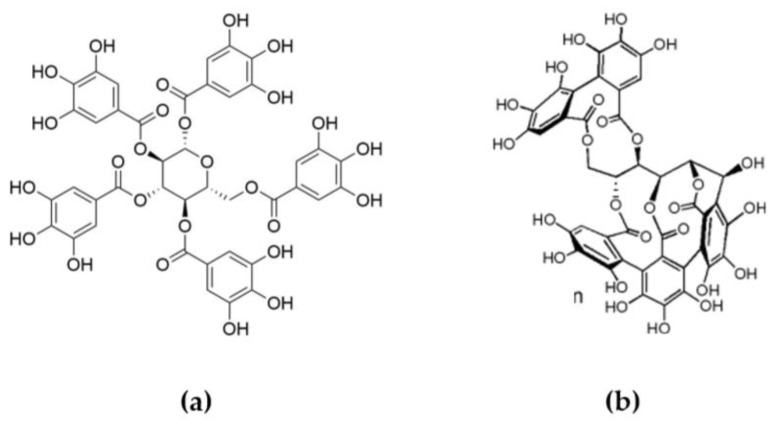
Typical molecules of hydrolysable tannins: (**a**) pentagalloyl glucose and (**b**) vescalagin.

**Figure 2:**
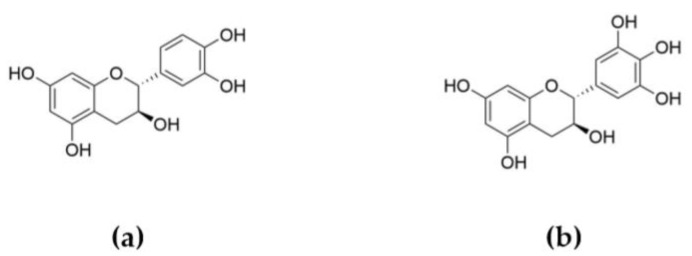
Typical constitutive units of condensed tannins: (**a**) (+)-catechin and (**b**) (+)-gallocatechin.

Tannins are a group of phenolic biopolymers and macromolecules widely distributed in plant cells [8,9]. There are two main classes of tannins: hydrolysable tannins, based on gallic or hexahydroxyphenic acids moieties connected to a central polyol or osidic core (Figure 1), and condensed tannins, polymers of flavonoid-based subunits (Figure 2). Considering their chemical structures and thermal properties provided by the aromatic rings, tannins present an interest for the development of FR additives.

In the last ten years, a particular focus has been the development of condensed tannins-based FRs [10,11,12]. The efficiency of mimosa tannins–boron preservatives for wood buildings has been studied [10,11]. It was found out that the use of tannins and tannins–boron additives provided significant increase of the time to ignition (TTI) (from 12 s to > 120 s) with a decrease of mass loss rate of scots pinewood. The application of condensed tannins as FRs additives for silk textiles has been previously reported [12]. The use of tannins allows flame retardancy of silk fabric to be improved. The textile treated with tannins exhibited a limiting oxygen index (LOI) above 27.0%, whereas the LOI of the untreated fabric was 24.7%.

The effectiveness of hydrolysable tannins as FRs was not considerably investigated until recently [13,14]. The treatment with tannic acid alone changed the thermal model of the pyrolysis and combustion of cotton and enhanced the char yield [13]. The addition of sodium hydroxide (SH) during the treatment contributed to the adsorption of tannic acid onto cotton. It also catalyzed the decarboxylation of tannic acid and the dehydration of cotton cellulose at lower temperatures. These chemical modifications induced a reduction of heat release capacity (HRC) by 82% (from 278 to 51 J (g K)^−1^) and an increase of the LOI values from 19.0 to 30.2% compared with that of the control cotton. However, more attention should be given to FRs based on hydrolysable tannins. Their aromatic rings and hydroxyl functions available for a chemical modification, as well as their abundance in plant biomass such as chestnut, tara, and oak trees [8], make them potential building blocks for bio-based FR agents.

**Figure 3:**
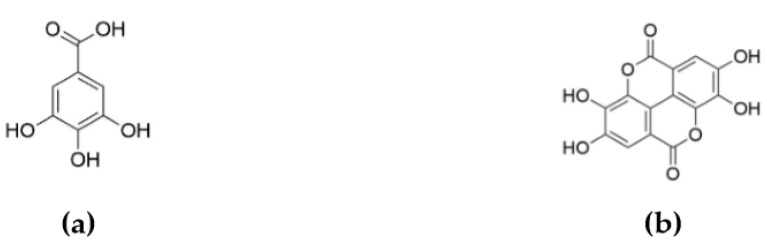
Structure of (**a**) gallic and (**b**) ellagic acids.

Hydrolysable tannins are usually subdivided in two groups: gallotannins and ellagitannins [8]. Gallotannins are polymers based on various numbers of galloyl units linked to a central polyol. Their acidic, alkali, or enzymatic hydrolysis leads to the release of gallic acid (GA) (Figure 3a). As for ellagitannins, they contain various numbers of hexahydroxydiphenoyl (HHDP) units, which after hydrolysis spontaneously dehydrate to ellagic acid (EA) (Figure 3b) [15]. Due to their biological activities in plants, investigation of their health properties and use as drugs, dietary supplements, or health care products has been undertaken. Nevertheless, both GA and EA can be employed for high added-value applications in specialty chemistry and polymeric material sectors thanks to their particular chemical structures. Moreover, phosphorus derivatives of GA have already shown their efficiency as biobased FR agents for the formulation of epoxy resin based on diglycidyl ether of bisphenol A (DGEBA) and 2-ethyl-4-methylimidazole [16]. Indeed, the incorporation of 15 wt.% of GA modified with diethylphosphite (DEP) induced a reduction of peak of heat release rate (pHRR) by 41% (from 692 to 411 W g^−1^) and an increase of LOI value by 21% (from 19.0 to 23.0%) compared to the neat epoxy resin.

Boron-based FRs are considered as an efficient harmless alternative to halogen-containing FRs in polymeric materials [17,18]. They act as a multifunctional FRs providing many useful properties: the promotion of the char formation, the stabilization of the char by developing glass barriers, and the suppression of smoke and carbon monoxide formation [19,20]. Usually, boric compounds are coated onto cellulosic products but they can also be employed in epoxy resins for improving their thermal properties. The use of boric acid (BA) in epoxy-diamine resin cured with hexamethylenediamine has been previously reported [21]. In this case, the temperature of 50 % weight loss of the epoxy resin containing 10 wt.% of BA was 15.8% higher than that for the neat epoxy polymer (425 °C as opposed to 367 °C). It was also previously reported that BA and its derivatives may be used to improve fire retardancy of phenolic compounds [19]. Therefore, considering the advantages, the grafting of boron on phenolic components should lead to an efficient biobased halogen-free FR for epoxy thermoset.

The present work reports the development of halogen-free FRs based on GA and EA phenolic acids as well as their borate forms, and a study of their impact on the thermal properties and fire behavior of a model epoxy thermoset. The resin was prepared by crosslinking of diglycidyl ether of bisphenol A (DGEBA) with isophorone diamine (IPDA).

## 2. Results and Discussion

### 2.1. Characterization of the FRs

#### 2.1.1. Chemical Characterization of the FRs

**Figure 4:**
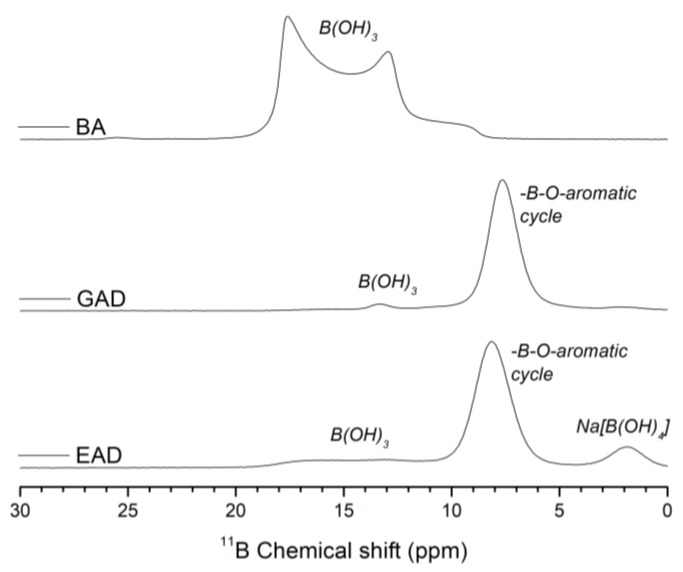
^11^B NMR spectrum of boric acid (BA), gallic acid derivatives (GAD), and ellagic acid derivatives (EAD).

Mass spectrometry (MS), ^11^B solid-sate nuclear magnetic resonance (NMR) spectroscopy and inductively-coupled plasma–atomic emission spectrometer (ICP–AES) analyses of the FR agents were performed. The results are reported in Figure 4 and Table 1. 

**Figure 5:**
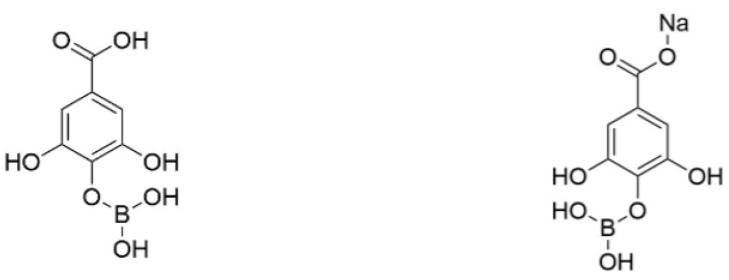
Putative structures of GAD deduced from MS and ^11^B NMR analyses.

**Gallic acid derivatives (GAD).** Gallic acid derivatives (GAD) were prepared by treatment of GA and BA at pH = 9 in the presence of SH. GAD were recovered by freeze-drying of the treatment solution. A sample of freeze-dried GAD powder was dissolved in methanol prior to ESI–MS analysis. The MS analysis showed the presence of various signals, of which two major ones were detected at m/z 241 and 263 in the negative ion mode (Table 1). The lowest m/z value was assigned to a gallic acid molecule linked through an oxygen atom of the phenolic hydroxyls to B(OCH_3_)_2_, while m/z 263 was attributed to the sodium adduct of the former molecule. The methylation of borate groups likely occurred in methanol prior to MS analysis but GAD are not originally methylated and the borate groups should be B(OH)_2_ in GAD. The ^11^B NMR spectrum of GAD confirmed the treatment of GA with BA (Figure 4). In fact, the spectrum displays a significant decrease of the signal at 13.9 ppm, which corresponds to three-coordinate boron sites of unreacted BA [22]. The signal for boron at 7.7 ppm is associated with a coordinate number of three, and it corresponds to the –B–O–aromatic cycle bonds. The putative structures of GAD are shown in Figure 5. The theoretical boron and sodium contents of GAD were calculated to be 4.23 and 5.37 wt.%, respectively (see the supplementary file). The boron and sodium contents of GAD measured by ICP–AES were quite close to the theoretical values (Table 1) and were equal to 3.69 ± 0.03 and 4.91 ± 0.02 wt.%, respectively.

**Figure 6:**
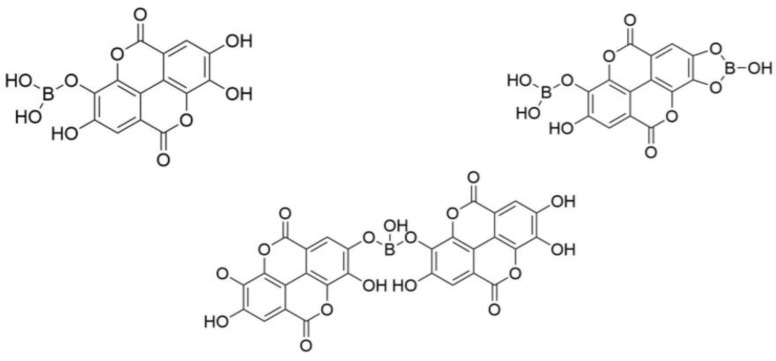
Putative structures of EAD deduced from MS and ^11^B NMR analyses.

**Ellagic acid derivatives (EAD).** EA was also borated by treatment with BA at pH = 9 in the presence of SH. As for GAD, the signals detected in ESI–MS analysis for EAD suggest the formation of methylated borate forms detected at m/z 373, 413, and 643 (Table 1). These m/z values were assigned to ellagic acid linked to B(OCH_3_)_2_ by one of the phenolic oxygen atoms, the same molecule containing a second borate group BOCH_3_ twice linked to two phenolic adjacent oxygen atoms and a dimer of ellagic acid linked by a borate group BOCH_3_ involving one phenolic oxygen atom of each ellagic acid unit, respectively. The ^11^B NMR analysis of EAD showed three different signals (Figure 4). The first one in the 18-13 ppm spectral region corresponds to unreacted BA. Since the structures of GA and EA are chemically close, the second signal at 8.1 ppm is associated with the -B-O–aromatic cycle bonds. The third signal at 1.9 ppm indicates the presence of four-coordinate boron, which corresponds to the product of the reaction between BA and SH—sodium tetrahydroxyborate Na[B(OH)_4_]—as it is shown in literature and confirmed by the laboratory tests [22]. From these results, Figure 6 represents the potential main chemical structures of EAD. The boron and sodium contents measured by ICP–AES analysis were quite close to the theoretical ones (Table 1) and equal to 3.51 ± 0.02 and 7.74 ± 0.03 wt.%.

#### 2.1.2. Thermal Characterization of GA and EA and their Boron Modified Entities

**Figure 7:**
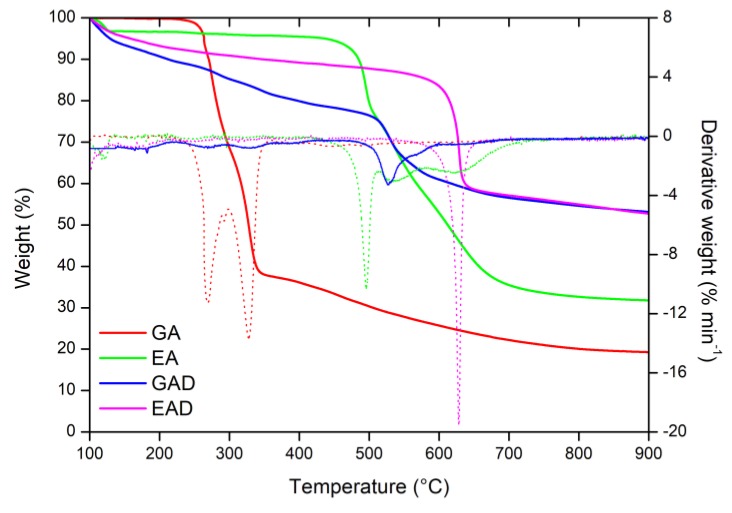
Thermograms and the corresponding differential curves of the FR agents at 10 °C min^−1^ under nitrogen flow.

The thermal decomposition and thermal stability of the FR agents were assessed by coupling thermogravimetric analysis with Fourier Transform InfraRed spectroscopy (TGA–FTIR). Figure 7 illustrates the thermogravimetric curves of the pure phenolic compounds and their synthesized derivatives under nitrogen flow. The decomposition temperatures (T_max_) as well as the char yield values at 600 °C (Char_600_) and 900 °C (Char_900_) are listed in Table 2.

It was observed that both unmodified phenolic compounds (GA and EA) showed a multistep degradation process.

**Gallic acid (GA).** The thermal decomposition of GA was composed of two main steps. It was stable up to around 230 °C at which point the first step of the thermal decomposition occurred with the mass loss at 269 °C (T_max1_). The second and major degradation step occurred at 327 °C (T_max2_). Carbon dioxide (CO_2_) was the main component of the off-gases for these two steps. It corresponds to the characteristic bands at 2359 and 2323 cm^−1^ (stretching vibrations) and 668 cm^−1^ (in-plane vibration). The formation of CO_2_ is principally associated with the decarboxylation of GA [23,24]. As described by Rama Mohan Rao et al. [25], water is also released during these two steps of decomposition. Above 350 °C, no other signals were detected. The char yield at 600 and 900 °C were equal to 25.7 and 19.3 wt.%, respectively.

**Ellagic acid (EA).** EA exhibited a more remarkable thermal stability than GA. It could be due to its specific chemical structure characterized by relatively stable central biphenyl bond and lactone groups (Figure 3b) [25,26]. The TGA–FTIR analysis suggested a three-step thermal decomposition process of EA. The weight loss at about 100 °C was mainly associated with the evaporation of water attached to EA. The first and major step started at around 400 °C and showed the maximum signal at 496 °C (T_max1_). The second and third steps were associated with the mass loss at 528 (T_max2_) and 630 °C (T_max3_), respectively. These steps were mainly characterized by CO_2_ release, but carbon monoxide CO with the characteristic bands at 2180 and 2107 cm^−1^ was also detected. These compounds are mainly ascribed to the pyrolysis of the lactone groups of EA [27,28]. No signal was observed above 650 °C. The char yield values at 600 and 900 °C were more important than those of GA—52.9 and 31.7 wt.%, respectively. The different charring abilities of GA and EA could be related to their chemical structures. In general, aromatic compounds show a formation of important carbon residue during the TGA test. EA contains two aromatic rings in its structure, while GA has just one. Thus, it could be assumed that EA better promotes the char formation than GA. In addition, EA shows more important carbon content than GA—56 against 49 wt.%, respectively.

**Boron derivatives of EA and GA (GAD and EAD).** The boration of the phenolic compounds allowed improving their thermal stability. For both GAD and EAD a progressive weight loss was observed from 100 °C up to their main decomposition step. This phenomenon was associated with the dehydroxylation of boron derivatives [29]. It was confirmed by the appearance of bands related to water in FTIR spectra. The major decomposition step occurs at 528 °C (T_max1_) for GAD and at 628 °C (T_max1_) for EAD, respectively. It was mainly ascribed to the pyrolysis of carboxylic and lactone groups. The main products related with this process, CO_2_ and CO, were detected in the off-gasses of the heating sample. No boron- and sodium-containing volatile compounds were detected. Therefore, it may be assumed that boron and sodium atoms mainly remain in the solid phase. In the case of GAD, it is noteworthy that the formation of a sodium carboxylate (see Figure 5) stabilizes the carboxylic group that decomposes 200 °C higher compared with GA. The carboxylate form should be predominant in GAD since only a small weight loss was observed at 300 °C (decarboxylation temperature of GA). The boron modification enhances the residue yield of the phenolic compounds. The char_600_ and the char_900_ values were equal to 60.7 and 53.1 wt.% for GAD and to 83.5 and 52.7 wt.% for EAD, respectively.

**Figure 8:**
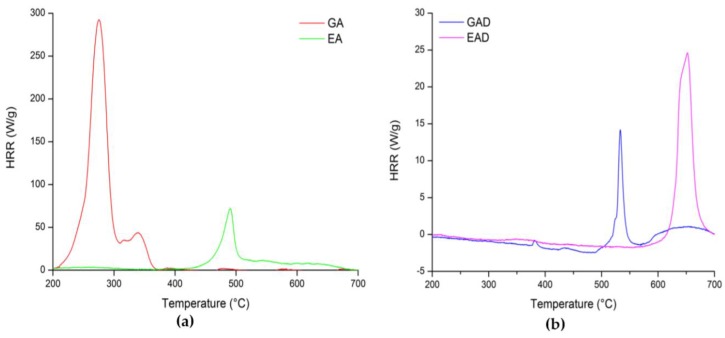
Pyrolysis/combustion flow calorimetry (PCFC) curves of (**a**) the phenolic compounds and (**b**) their boron derivatives at 1°C·s^−1.^

To assess the flame retardant potential of the additives, pure phenolic compounds (GA and EA) and their boron derivatives (GAD and EAD) were characterized by pyrolysis/combustion flow calorimetry (PCFC). The heat release rate (HRR) curves of the FR systems are shown in Figure 8. Table 3 summarizes the values of pHRR, temperature of pHRR (T_peak_), total heat release (THR), and theoretical and experimental effective heat of combustion (EHC_theo_ and EHC_exp_, respectively). All the results obtained by the PCFC analyses were in accordance with the TGA measurements (Table 2).

It was confirmed that GA decomposes in two main steps, whereas EA essentially does it in three steps (the one at about 100 °C is not observed on the PCFC curve since it is related to the release of water). It was observed that GA is the most exothermic compound with the pHRR equal to 293 W g^−1^ and the THR equal to 12.4 kJ g^−1^, respectively, while EA shows considerably lower pHRR and THR values for an organic molecule (73 W g^−1^ and 2.9 kJ g^−1^, respectively). As is shown in Table 3, GA exhibits similar EHC_theo_ and EHC_exp_ values—14.8 and 15.9 kJ g^−1^, respectively. Moreover, the ratio THR/EHC_theo_ is approximately 80%, indicating that the THR decrease is almost proportional to the char yield (circa 20%). As for EA, its EHC_exp_ value is much lower than its EHC_theo_—4.5 and 16.0 kJ g^-1^, respectively. The ratio THR/EHC_theo_ is 18%, meaning that the THR decrease is much higher that the char yield. However, thermochemistry calculations do not take into account the energy stored in the char. To correct this, the EHC_theo_ should be calculated again by considering the char composed only of carbon (${\mathrm{EHC}}_{\mathrm{theo}}^{\mathrm{char}})$. The ${\mathrm{EHC}}_{\mathrm{theo}}^{\mathrm{char}}$ value of EA was calculated according to the method proposed by Dorez et al. [30]. The ${\mathrm{EHC}}_{\mathrm{theo}}^{\mathrm{char}}$ value of EA was much closer to the EHC_theo_ one and equal to 15.7 kJ g^−1^. Therefore, the low EHC_exp_ and THR values of EA are mainly due to the charring ability of the compound that enables storage of energy in the char [30].

The PCFC measurements also confirmed the improvement of GA and EA thermal characteristics by their functionalization. In fact, GAD and EAD exhibited the pHRR equal to 10 and 25 W g^−1^, respectively, while the THR values were equal to 0.3 kJ g^−1^ for GAD and 0.5 kJ g^−1^ for EAD, respectively. In addition, GAD and EAD showed a decrease of the EHC_exp_ values compared to the EHC_exp_ of GA and EA—0.6 and 1.0 kJ g^−1^, respectively. The EHC_theo_ values of GAD and EAD cannot be calculated because of their non-uniform chemical structure. That is why the EHC_theo_ and EHC_exp_ values of GAD and EAD cannot be compared.

### 2.2. Influence of FR Systems on Epoxy Properties

The adhesion between the epoxy matrix and the FR additives as well as the influence of additives on epoxy crosslinking are key factors that may affect the thermal and mechanical properties of FR/epoxy systems. Therefore, dispersion within epoxy thermoset was studied through SEM observations, and differential scanning calorimetry (DSC) experiments were carried on.

#### 2.2.1. Dispersion of FR Agents within Epoxy Resin

**Figure 9:**
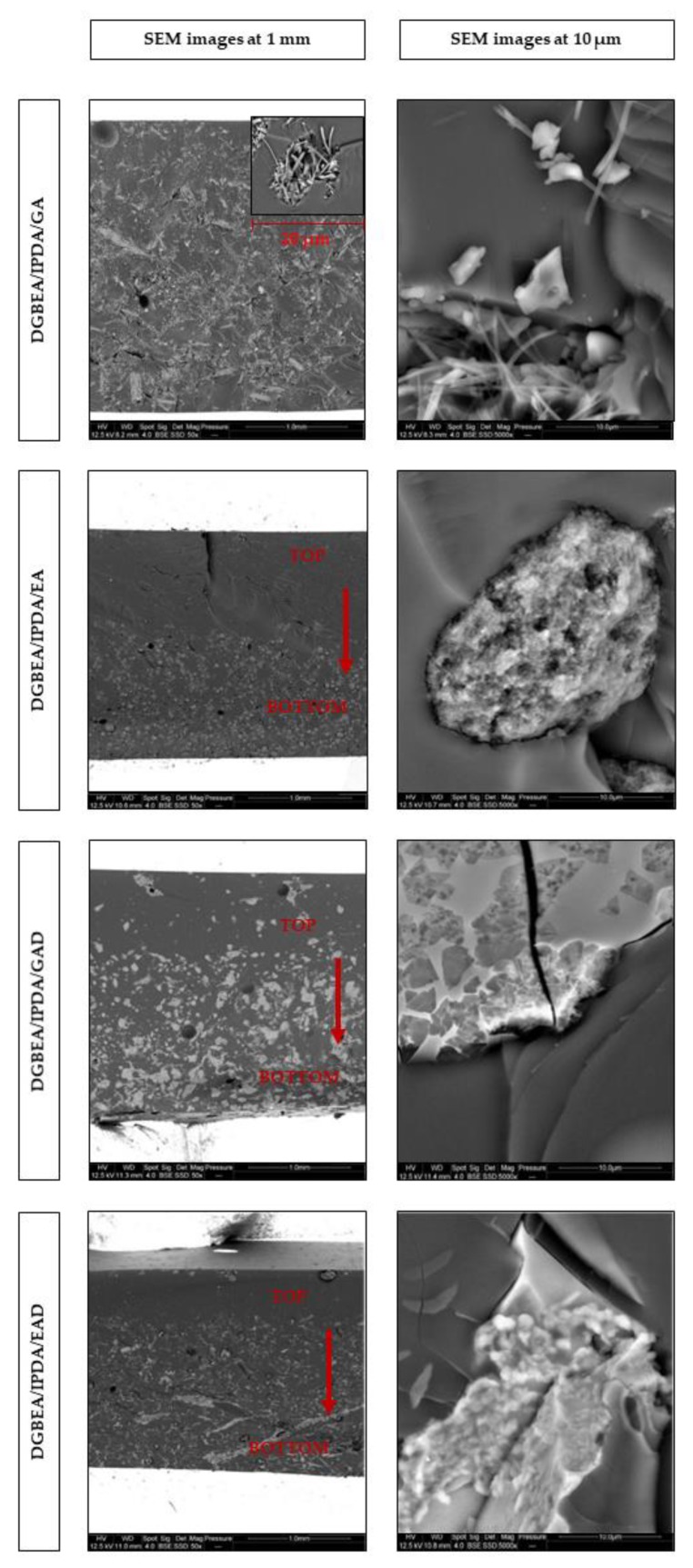
The SEM images of the formulated epoxy resins.

The SEM observations of the formulated epoxy thermosets are illustrated in Figure 9.

Non-uniform dispersion of the pure phenolic components into the epoxy matrix was clearly observed. The agglomeration process of acicular particles of GA and spherical particles of EA into DGEBA/IPDA/GA and DGEBA/IPDA/EA thermosets, respectively, was noticed. This can be explained by (1) mixing parameters such as speed and time of dispersion, (2) some adhesion forces (interlocking, electrostatic, and/or van der Waals forces) holding the particles together [31], and (3) the low wettability of the phenolic particles by the liquid epoxy resin, which induces the lack of interactions (observed in the SEM images at 10 µm) [32]. Moreover, a sedimentation phenomenon was evidenced when observing the sample cross sections especially for the DGEBA/IPDA/EA system. The EA content was higher at the bottom area of the sample than at the top one (observed in the SEM image at 1 mm). Sedimentation may occur during the early stage of curing when a decrease in epoxy resin viscosity is occurring. At this moment, highly agglomerated particles of EA settle into the epoxy resin.

A chemical treatment is usually able to prevent such an agglomeration by changing interfacial bondings with the epoxy matrix [32]. Nevertheless, it was not the case for GAD and EAD particles in DGEBA/IPDA/GAD and DGEBA/IPDA/EAD resins, respectively. It is likely that aggregates were formed during the freeze-drying step after chemical treatment. Further works are required to reduce the particle size before incorporation into epoxy. Nevertheless, it can be noticed that GAD and EAD particles exhibited better affinity with the epoxy matrix than GA and EA (observed in the SEM images at 10 µm).

#### 2.2.2. Thermal Properties of the FR Epoxy Resins

**Figure 10:**
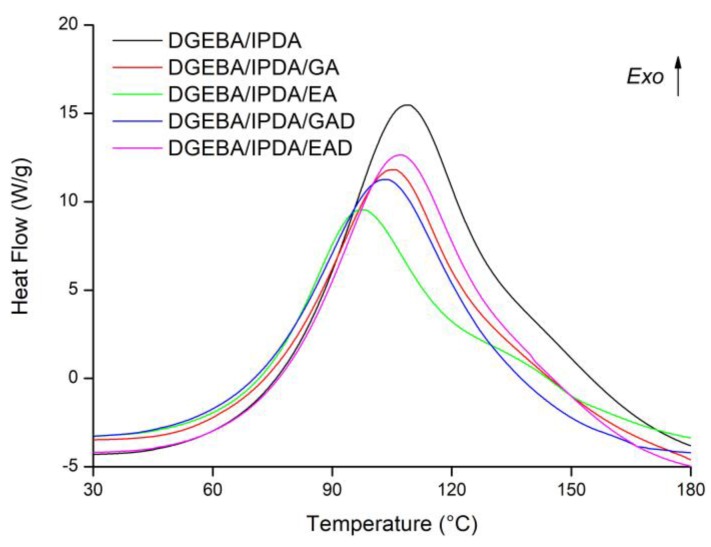
Differential scanning calorimetry (DSC) thermograms at 10 °C min^−1^ of uncured bisphenol A diglycidyl ether and isophorone diamine (DGEBA/IPDA) mixtures in the presence or not of FRs.

DSC analyses were performed on the non-cured epoxy resins to observe the influence of FR systems on epoxy crosslinking. The glass transition temperature was determined after the resins were cured according to the procedure described in Section 3.3. The results are reported in Figure 10 and Table 4.

During a first heating at 10 °C min^−1^, the neat epoxy resin showed an ideal exothermic peak (T_p_) at 109 °C. Its total heat of reaction (ΔH) was equal to 431 J g^−1^ corresponding to 96 kJ ee^−1^. The cured DGEBA/IPDA thermoset exhibited a glass transition temperature (T_g_) equal to 144 °C. These results are consistent with those previously reported [33].

The incorporation of the phenolic-based additives such as GA, GAD, and EAD into DGEBA/IPDA resin does not lead to significant changes in thermal characteristics of the material (Table 4). No signal corresponding to the interaction between the additives and the epoxy resins was observed. The T_p_ values of these resins were slightly reduced compared to the neat resin and ranged between 105 and 108 °C. The lowest and the highest values correspond to DGEBA/IPDA/GAD and DGEBA/IPDA/EAD thermosets, respectively. The ΔH values calculated by gram of material were logically reduced by about 10% with the incorporation of 10 wt.% of the additives compared to the neat thermoset. The enthalpies of reaction calculated by epoxy equivalent (ee) were quite close to that of the neat resin except for DGEBA/IPDA/EA. It allows concluding that GA, GAD, and EAD do not influence the cure kinetic of the epoxy resin. On the contrary, the use of EA as additive affects thermal properties of the epoxy thermoset. An exothermic peak at 99 °C with a shoulder between 120 and 150 °C was observed for the DGEBA/IPDA/EA thermoset (Figure 10). The enthalpy of reaction was decreased by 7 %, compared to the DGEBA/IPDA thermoset. Therefore, it could be assumed that EA affects the curing kinetic of the epoxy thermoset by reacting as a hardener [34]. It was noticed that the T_g_ values of the additive-containing resins were reduced compared to the neat epoxy resin (Table 4). The T_g_ values of the DGEBA/IPDA/GA, DGEBA/IPDA/EA, and DGEBA/IPDA/EAD resins were equal to 127, 137, and 110 °C, respectively, whereas DGEBA/IPDA/GAD showed a T_g_ equal to 108 °C. The reduction of the T_g_ values could be due to the non-uniform dispersion of the additives within the epoxy resin and the important agglomeration of the particles (the case of DGEBA/IPDA/GAD) as already observed by the SEM analyses (Figure 9).

**Figure 11:**
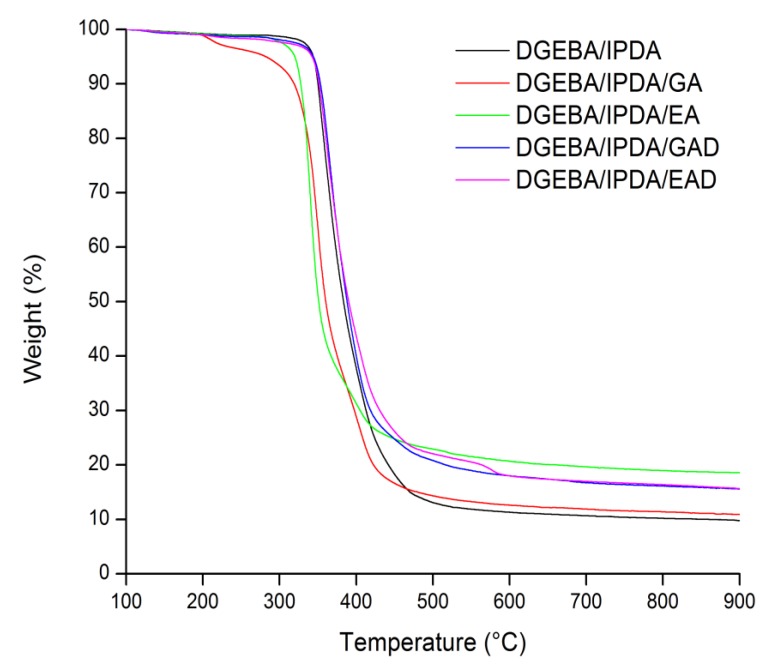
Thermograms of epoxy resin and flame retarded systems at 10 °C min^−1^ under nitrogen flow.

TGA–FTIR analysis was carried out to assess the impact of the pure phenolic compounds (EA and GA) and their derivatives (EAD and GAD) on the thermal stability and charring ability of DGEBA/IPDA resin. TGA curves under nitrogen are illustrated in Figure 11. The thermal characteristics are listed in Table 5.

The GA- and EA-containing epoxy thermosets showed a premature onset of decomposition compared to the unfilled epoxy resin. The T_max_ was reduced from 370 °C for DGEBA/IPDA, to 349 and 338 °C for DGEBA/IPDA/GA and DGEBA/IPDA/EA, respectively. Two phenomena could be responsible for this reduced thermal stability. The first one is the early stage of decomposition of the pure phenolic compounds. The effect is particularly noticeable for the GA-containing thermoset. The onset of weight loss at about 200 °C corresponds to the beginning of thermal decomposition of the pure phenolic additive (Figure 7). A second phenomenon influencing the thermal stability of the thermosets may be the pro-degradant effect of hydroxyl group bonds by the phenolic compounds. Such an effect has already been observed when incorporating lignin into polylactic acid (PLA), leading to the premature degradation of the polymer matrix [35]. The char yields of DGEBA/IPDA were improved by using the pure phenolic components as additives. The char_600_ values increased from 11.3 wt.% for DGEBA/IPDA to 12.6 and 20.7 wt.% for DGEBA/IPDA/GA and DGEBA/IPDA/EA, respectively. It was observed that the theoretical (calculated using the additive law) and experimental char yields at 900 °C of DGEBA/IPDA/GA thermoset were almost the same - 10.7 and 10.9 wt.%, respectively. Whereas the theoretical char_900_ value of DGEBA/IPDA/EA was lower than the experimental one—12.0 against 18.6 wt.%, respectively, it could be explained by some reactions between the particles of EA and the epoxy matrix (observed by the DSC analysis). The formed products seem to promote the charring ability of the thermoset.

Concerning the GAD- and EAD-containing epoxy thermosets, they exhibited similar thermal stability to the neat epoxy resin. The T_max_ values of DGEBA/IPDA/GAD and DGEBA/IPDA/EAD were equal to 367 and 371 °C, respectively. It is assumed that the pro-degradant effect of hydroxyl groups from GA and EA was screened after functionalization. An increase of the char yield up to 18.0 wt.% was observed for both thermosets. The theoretical char yields at 900 °C of the epoxy resins were close to the experimental ones: 14.1 against 15.6 wt.% for DGEBA/IPDA/GAD and 14.1 against 15.8 wt.% for DGEBA/IPDA/EAD, respectively. These results allowed considering that GAD and EAD do not promote additional charring of the DGEBA/IPDA matrix.

The off-gases of the DGEBA/IPDA thermosets analyzed by FTIR correspond to those previously reported [36]. The FTIR spectrums of the off-gases showed bands of C=O (1751 cm^−1^), C_Ar_—O groups of phenol (1260 and 1176 cm^−1^), C_Ar_=C_Ar_ (1603 and 1507 cm^−1^), C_Ar_–H (3057, 746, and 686 cm^−1^), and OH–phenolic groups of bisphenol A (3649 cm^−1^). The decomposition products as CO_2_ (2379 and 2311 cm^−1^), CO (2180 and 2107 cm^−1^), ammonia NH_3_ (965 and 931 cm^−1^), methane CH_4_ (3018 cm^−1^), and free H_2_O (3756 and 1595 cm^−1^) were also detected.

#### 2.2.3. Fire Behavior of the FR Epoxy Resins

**Figure 12:**
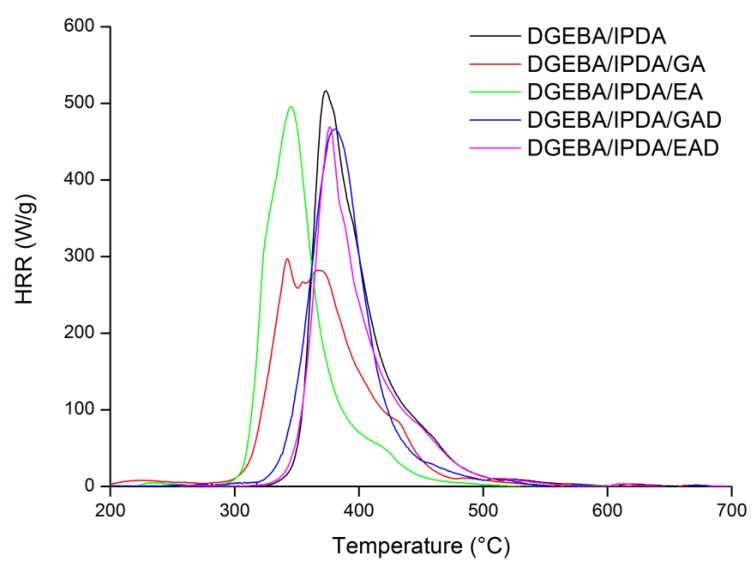
PCFC curves of the DGEBA/IPDA based flame retarded systems at 1 °C s^−1.^

Thermogravimetric analysis can be completed by the study of the fire behavior of the formulated FR epoxy thermosets using PCFC and cone calorimeter measurements. Figure 12 illustrates the HRR curves of the epoxy thermosets obtained by PCFC measurements. The PCFC characteristics are listed in Table 6. The PCFC results are in accordance with the TGA measurements for all the studied samples (Table 5). Hence, GA and EA containing epoxies exhibit pHRR temperatures lower than those of the reference and the boron derivatives containing epoxies. This confirms the destabilizing effect of the pure phenolic compound onto the polymer matrix.

The neat epoxy resin shows high pHRR and THR values during PCFC test—525 W g^−1^ and 28.4 kJ g^−1^, respectively. Similar results were previously reported [37]. A decrease in these values was observed after adding the pure phenolic compounds into the epoxy system. The pHRR values of the GA- and EA-containing epoxy thermosets decreased respectively to 292 and 471 W g^−1^. Regarding EA containing epoxy, this corresponds to a 10% reduction in good agreement with an additive mixing law. On the opposite, the DGEBA/IPDA/GA resin exhibits a 45% pHRR reduction, i.e., much higher than expected from the mixing law. It allows suggesting that this pure phenolic compound may influence the combustion pathway of the epoxy thermoset. The THR values of the GA- and EA-containing resins were reduced by about 10% compared to the uncharged epoxy thermoset and were equal to 25.8 and 25.9 kJ g^−1^, respectively. No significant variation was observed for the EHC_exp_ values of the formulated thermosets. They were equal to 29.3 and 32.3 kJ g^−1^ for the GA- and EA-containing thermosets, respectively, compared to 31.8 kJ g^−1^ of the neat epoxy resin.

GAD and EAD led to the improvement of the fire behavior of the epoxy resin. The pHRR, THR, and EHC_exp_ values of DGEBA/IPDA/GAD and DGEBA/IPDA/EAD were decreased in proportions compliant with the mixing rule. Hence, no specific interactions with the polymer matrix were highlighted by this method.

**Figure 13:**
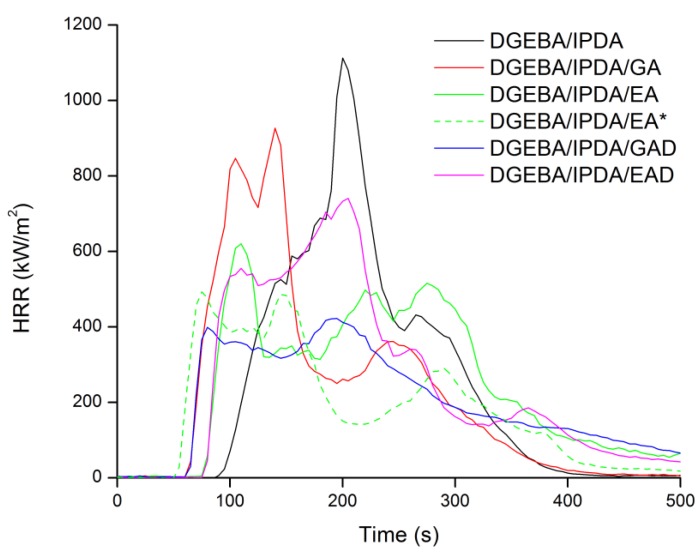
Heat release rate (HRR) curves of the formulated epoxy resin studied in a cone calorimeter at 35 kW m^−2.^.

It is well known that the PCFC test is not sensitive to physical effects while both chemical and physical actions are effective in cone calorimetry. Therefore, the fire behavior of the epoxy resins was also investigated using a cone calorimeter. It provides key information about flame retardant properties of materials such as time to ignition (TTI), HRR, pHRR, THR, and char content. The results of cone calorimeter test are summarized in Figure 13 and Table 7. A sedimentation issue was observed by SEM measurements for all the samples (Figure 9). In order to assess the fire behavior under the least favorable conditions, samples were exposed to the incident heat flux by the side with lower additive content, except DGEBA/IPDA/EA for which both sides were exposed.

**Figure 14:**
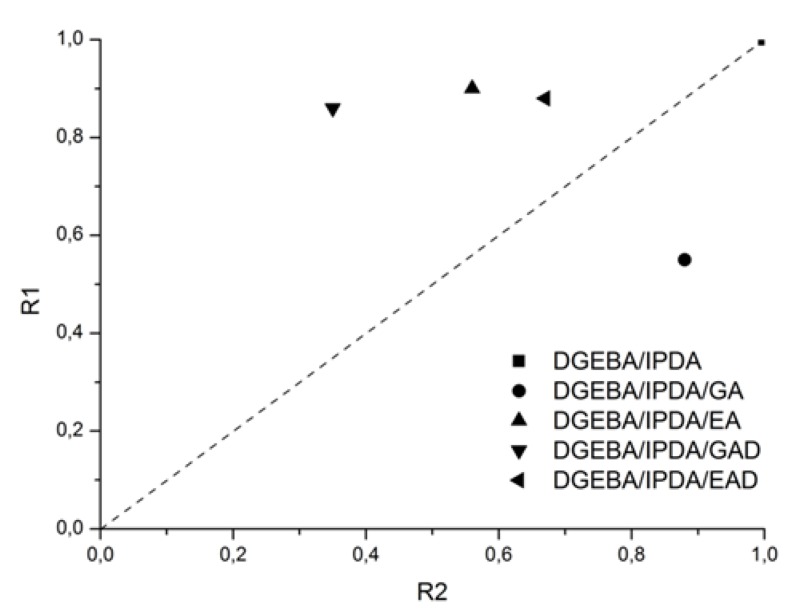
R1 vs. R2 plots for the DGEBA/IPDA thermosets.

According to the literature [36] and cone calorimeter tests, DGEBA/IPDA exhibits a high pHRR and a low carbon residue content—1116 kW m^−2^ and 3.9 wt.%, respectively. The addition of the phenolic compounds and their derivatives in DGEBA/IPDA leads to the evolution of its burning behavior. The use of the pure phenolic additives induced a decrease of TTI values from 90 s for DGEBA/IPDA to 66 and 80 s for the GA- and EA-containing epoxy resins, respectively. These variations in TTI values are in good agreement with the lower thermal stability of these compositions as shown by TGA and PCFC. It should be noted that a decrease of TTI was also observed in the presence of the boron derivatives GAD or EAD despite their good thermal stability as observed in TGA. In a general way, the incorporation of 10 wt.% of the phenolic compounds or their derivative in DGEBA/IPDA allows reducing both the pHRR and THR. In the case of DGEBA/IPDA/GA, the pHRR value is reduced only by 12% while for DGEBA/IPDA/EA, the decrease is much more significant (by 44%). The most remarkable effect was obtained with GAD that induces a 65% reduction of pHRR while the results for EAD were a little bit disappointing with only 31% reduction of pHRR. In order to evaluate the influence of the additive dispersion in the epoxy matrix, the sample DGEBA/IPDA/EA was exposed to the incident heat flux by the side with the higher EA content referred to as DGEBA/IPDA/EA*. It was observed that TTI was decreased to 55 s. This result was associated with the early decomposition of this composition as observed by TGA. Nevertheless, the pHRR value was significantly reduced to 492 kW m^−2^ which corresponds to a decrease by 21 % compared to the DGEBA/IPDA/EA sample. It leads to the conclusion that the non-uniform dispersion of additives in the epoxy matrix may affect the fire behavior of material. A uniform dispersion of the additives in the epoxy matrix should be achieved to ensure a better efficiency as FRs. Apart from the decrease of pHRR, it is noteworthy that the use of phenolic compounds (GA, EA, EA*, and GAD) induces a change in the HRR curve shape. Hence, for these compositions, the pHRR happens rapidly after ignition and then HRR tends to decrease. This behavior is typical of thick charring materials according to the classification of Schartel and Hull [38]. It means that quickly after ignition a protective layer is formed at the sample surface that acts as heat shield for the underlying polymer thus reducing its pyrolysis rate and consequently the heat release rate as well. With a view to evaluate this barrier effect in the epoxy resin, PCFC and cone calorimeter measurements can be used according to the method reported by Sonnier et al. [39]. This method consists in plotting two parameters R1 (Y-axis) vs. R2 (X-axis), where R1 is the ratio between HRC in PCFC of the additive-containing resin and HRC in PCFC of the neat resin and R2 is the ratio between pHRR in the cone calorimeter of the additive-containing resin and the pHRR in the cone calorimeter of the uncharged resin. At 1 K s^−1^, the HRC is equal to the pHRR. The method relies on the assumption that the barrier effect is only operative in the cone calorimeter but not in PCFC where samples are supposed to be at a uniform temperature. Consequently, the occurrence of a barrier effect can be evidenced by a deviation from the R1 = R2 line. As observed in Figure 14, the values corresponding to epoxy containing EA, GAD, and EAD are located above the line R1 = R2 (which corresponds to a similar decrease of pHRR in the cone calorimeter and of HRC in PCFC). It indicates that the decrease of pHRR in the cone calorimeter is higher than the decrease of HRC in PCFC highlighting a significant barrier effect. The strongest barrier effect was observed for DGEBA/IPDA/GAD. These results are consistent with the residual mass after the flame out which is the highest for the GAD containing epoxy (13.7 wt.%).

**Figure 15:**
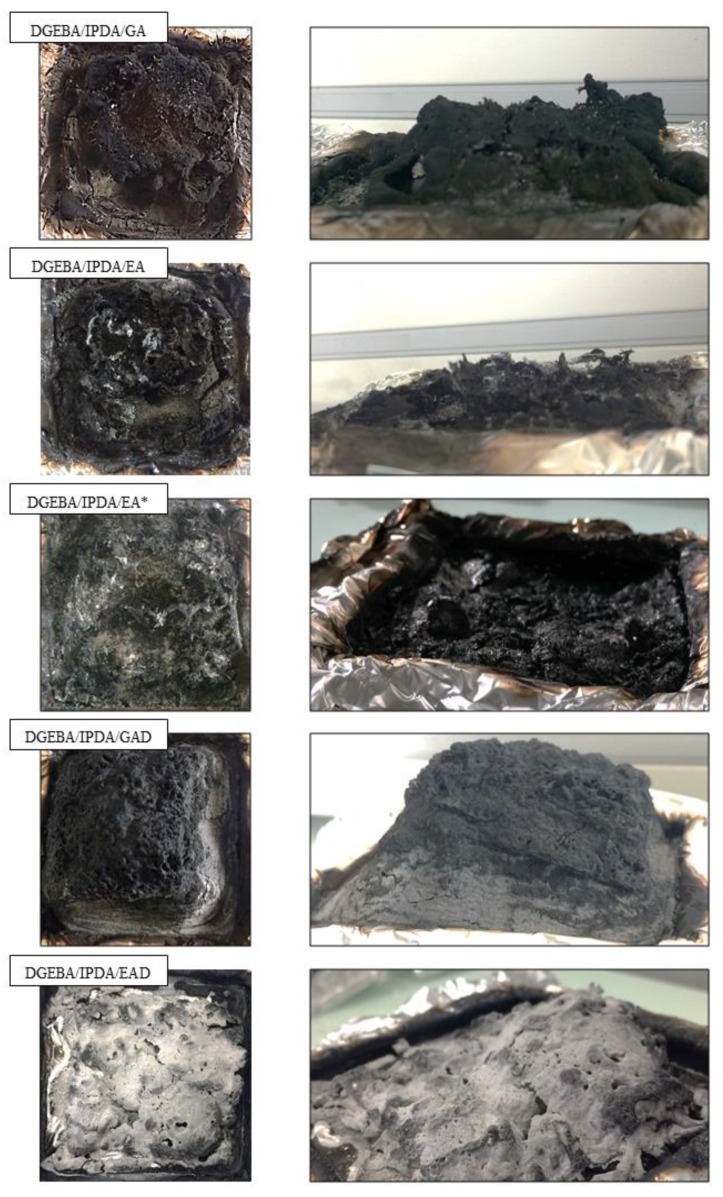
Cone calorimeter residues of the prepared thermosets.

The barrier effect can also be grasped by observing the cone calorimeter test residues. Figure 15 shows that epoxies containing GA and EA exhibit some charring, however in both cases the cohesion of the residue seems very poor which may explain that the heat release rate is not really under control. On the opposite, GAD containing epoxy exhibits a cohesive residue. Moreover, this residue is significantly expanded (Figure 15) which emphasizes its efficiency as a heat shield. The grey color at the bottom of the residue may indicate that boron plays a role in its cohesion as a glassy binder. This assumption is corroborated by the fact that boron remains the residue as shown in Table 7. A similar behavior was expected in the case of EAD. Figure 15 shows that the boron derivative seems to impart some cohesion to the residue. However, the charring effect appears as negligible, the residue being almost entirely grey. Moreover, no expansion of the layer was observed during the combustion. In addition, results of ICP analysis (Table 8), performed on the char formed during the combustion confirm that GAD and EAD act in the condensed phase. In fact, the boron and sodium contents of the epoxies char residues measured by ICP–AES analysis were quite close to the theoretical ones, which were calculated considering the amount of the inorganic components in the GAD and EAD containing materials constant before and after fire testing. This result clearly indicates that GAD and EAD act mainly in condensed phase.

## 3. Materials and Methods

### 3.1. Materials

**Figure 16:**
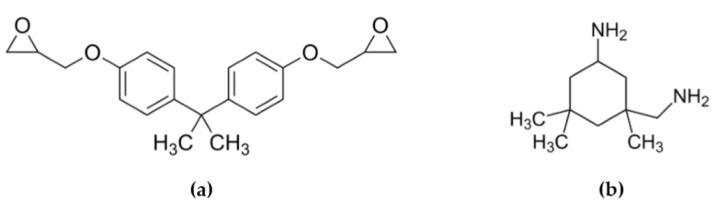
Structure of (**a**) DGEBA and (**b**) IPDA.

Ellagic acid labeled EA (purity 97%) was purchased (Alfa Aesar, Kandel, Germany). Gallic acid labeled GA (purity 97.5%) was supplied (Sigma–Aldrich, Saint Louis, MO, USA). Boric acid labeled BA (≥99%) was provided (GPR Rectapur, Leuven, Belgium). Sodium hydroxide abbreviated by SH (≥99%) was supplied(Roth, Karlsruhe, Germany). The conventional petroleum-sourced epoxy monomer used was diglycidyl ether of bisphenol A (DGEBA) (Figure 16a) and was supplied with an epoxy equivalent weight (EEW) of 177 g eq^−1^ (Sigma Aldrich, Kandel, Germany). The cycloaliphatic cross-linking agent isophorone diamine (IPDA) (Figure 16b) was supplied with an amine equivalent weight (AEW) of 42 g eq^−1^ (Sigma Aldrich, Steinheim, Germany).

### 3.2. Boratation of Phenolic Compounds

A two-step procedure was applied.

The first step consisted in the preparation of BA and SH solution. Fifty milliliters of 0.2 M aqueous solution of BA and 21.4 mL of 0.2 M aqueous solution of SH were mixed in a 100 mL beaker. The mixture was stirred vigorously for 5 min at room temperature and then stored at 4 °C until use. The pH of the solution was 9.

The second step corresponded to the functionalization. A 25 mL single-necked round-bottomed flask equipped with a magnetic stirring bar was charged with phenolic compound and aqueous solution of BA and SH. The pH of the solution was adjusted to 9 with 0.2 M aqueous solution of SH and then heated for 3 h at 50 °C under vigorous stirring. After that, the solution was freeze-dried over 48 h.

The formation of boron complexes of the phenolic compounds was supposed under the conditions described above. The hydroxyl groups of phenolic compounds in the ortho-position help a complex formation with BA [40]. However, the presence of a carboxyl group (electron-withdrawing group) is expected to weaken the boron complex formation [40]. Freeze-drying was applied to the treatment solution in order to prevent thermal decomposition of the synthesized boron-based phenolic derivatives. 

Table 9 summarizes information about the boration of the phenolic compounds.

### 3.3. Epoxy Resin Preparation

Different chemicals are under liquid forms (DGEBA and IPDA) while other are under solid forms (FR systems). In order to obtain a homogeneous dispersion, GA and EA as well as GAD and EAD were firstly introduced in DGEBA by manual stirring. The prepared mixture was then heated for 15 min at 80 °C. The curing agent–IPDA–was then added in a 1:1 molar ratio of epoxy group to active H of amine. The system was stirred manually for 5 min. The mixture was cross-linked in a silicon mold to the following temperature program: 4 h at 80 °C and 1 h at 150 °C under inert atmosphere.

Table 10 summarizes different epoxy resins formulated in this study.

### 3.4. Chemical Characterization

#### 3.4.1. Mass Spectrometry

Mass spectrometry (MS) analyses were performed on a Ion trap mass spectrometer with an electrospray ionization (ESI) interface (Bruker Daltonics, Wissembourg, France) operating in negative ion mode. The samples of additives were dissolved in methanol and then filtered through a polymer membrane with a pore diameter of 0.45 µm. The injection of samples was made by syringe. The following conditions of the ESI interface were used: drying gas flow, 8.0 L min^−1^; nebulize pressure, 3 bars; gas drying temperature, 200 °C; capillary voltage, 5000 V; fragmentor voltage, 1 V.

#### 3.4.2. ^11^B solid-state Nuclear Magnetic Resonance Analysis

^11^B solid-state nuclear magnetic resonance (NMR) analyses were performed on an Avance III 600 MHz spectrometer (Bruker, Wissembourg, France) operating with ^11^B frequency of 192.4 MHz. A magic-angle spinning (MAS) rate of 20 kHz was used for all experiments. The samples were packed into a 4 mm outer diameter ZrO_2_ rotor using a double resonance probe.

#### 3.4.3. Elementary Analysis

Boron and sodium concentrations were determined by inductively-coupled plasma–atomic emission spectrometer (ICP–AES, SGS Miltilab, France). Calibration curves were established by employing the certified samples of known boron and sodium concentrations. Peak intensity was converted into boron weight percentage using the calibration curves. All samples were analyzed three times. The calculations were based on a dry weight of samples.

### 3.5. Thermal Characterization

#### 3.5.1. Thermogravimetric Analysis Coupled with Infrared Spectroscopy

Thermogravimetric analysis (TGA) measurements were obtained on a thermal analysis system Setsys Evolution (Setaram) (Setaram, Caluire, France). The initial weight of each tested sample was about 10 mg. The samples were heated from 30 to 900 °C at a heating rate of 10 °C min^−1^ under nitrogen atmosphere. The degradation temperatures (T_max_) and the char yields at 600 °C (Char_600_) and 900 °C (Char_900_) were determined for each sample. The theoretical the char content at 900 °C (${\mathrm{Char}}_{900}^{\mathrm{th}}$) for the thermosets was calculated according to the additive law of mixtures:

(1) $$Char_{900}^{th}=fChar_{ad}^{exp}+\left(1-f\right)Char_{ep}^{exp}$$

where ${\mathrm{Char}}_{900}^{\mathrm{th}}$ is the theoretical char yield of the additive-containing epoxy resin at 900 °C, wt.%; f—the mass fraction of the additive (equal 0.1 for all formulated thermosets); ${\mathrm{Char}}_{\mathrm{ad}}^{\exp}$—the experimental char yield of the additive at 900 °C, wt.%; ${\mathrm{Char}}_{\mathrm{ep}}^{\exp}$—the experimental char yield of the uncharged epoxy resin at 900 °C, wt.%.

A Fourier transform infrared spectrometer (FTIR) Antaris IGC (Thermo Fisher Scientific, Illkirch, France) was connected to the previous thermal system. The temperatures of the transfer line and the gas cell were fixed at 210 °C. The FTIR spectrometer was settled to collect 8 interferograms at a resolution of 8 cm^−1^ in the spectral range of 4000–400 cm^−1^. The evaluation of the gas concentration during the test was measured using OMNIC© software (v. 9.2) from Thermo Fisher Scientific.

#### 3.5.2. Differential Scanning Calorimetry Analysis

Differential scanning calorimetry (DSC) measurements of samples were carried out under nitrogen atmosphere using a DSC Q200 (TA Instrument, Guyanourt, France). The thermal properties were analyzed at 10 °C min^−1^ between 30 °C and 250 °C to observe the exothermic temperature peak (T_p_), the total heat of reaction (ΔH), and the enthalpy of reaction of the uncured samples and the glass transition temperature (T_g_) of the cross-linked thermosets determined as the inflexion value in the heat capacity jump. The uncured samples were tested in duplicate, while the cures ones were tested in triplicate.

### 3.6. Characterization of Fire Properties

#### 3.6.1. Pyrolysis Combustion Flow Calorimetry

Pyrolysis combustion flow calorimetry (PCFC) analyses of materials were performed using a FAA Micro Calorimeter from Fire Testing Technology (FTT, West Sussex, UK). About 2 mg of sample was placed in the pyrolyzer, undergoing an increase of temperature from 100 to 750 °C at 1 °C s^−1^ under nitrogen flow. Then, decomposition gases were sent to a combustor where they were heated at 900 °C under air flow (N_2_/O_2_ = 80/20). In these conditions, combustion was considered to be complete. Heat release rate (HRR) value was calculated by oxygen depletion according to Huggett’s relation [41]. The total heat released (THR) was determined by integration of PCFC curves. The experimental effective heat of combustion (EHC_exp_) represents the released heat by mass loss and is calculated as a ratio between THR and mass loss measured using TGA. The theoretical effective heat of combustion (EHC_theo_) of the pure phenolic compounds was also calculated [30]. For the case of complete combustion, a chemical compound containing carbon, hydrogen, and oxygen undergoes oxidation that leads to carbon dioxide and water release according to the following equation:

(2) $$C_{c}H_{h}O_{m}+\left(c+\;\frac{h-2m}{4}\right)O_{2}\to cCO_{2}+\;\frac{h}{2}H_{2}O$$

Conforming to Huggett’s relation, the heat released by the combustion is 13.1 kJ per gram of consumed oxygen [41]. Therefore, EHC_theo_ of organic compounds can be calculated. All samples were analyzed in duplicate.

#### 3.6.2. Cone Calorimeter

Fire behavior of the epoxy resins was also studied using a cone calorimeter (Fire Testing Technology) according to the standard ISO 5660 (sample dimensions 100 × 100 × 4 mm^3^). The samples were exposed to an irradiance of 35 kW m^−2^ in the presence of a spark igniter to force the ignition. The peak of heat release rate (pHRR), the time to ignition (TTI), the total heat release (THR), and the residue content were measured. All blends were done in triplicate and results were averaged.

### 3.7. Morphological Characterization

A scanning electron microscope (FEI Quanta 200 ESEM, Felmi-Zfe, Graz, Austria) was used to observe the dispersion of the FRs in the epoxy resin. Blended samples were prepared using cryo-fractured cross-sections and analyzed under high vacuum at a 12.5 V with a working distance of 10 mm.

## 4. Conclusions

This paper reported the alternative using of bio-sourced phenolic compounds such as gallic and ellagic acids and their boron- and sodium-functionalized forms as FR agents in the epoxy resin. It was observed that EA may influence the cure kinetic of the epoxy thermoset, whereas the other additives act as invert additives. The incorporation of the FR agents caused the reduction of the glass transition temperature from 144 °C for the neat epoxy resin to 54 °C for the GAD-containing thermoset. This effect was mainly correlated to the low compatibility of the additives with the epoxy matrix as well as to their non-uniform dispersion and agglomeration within the epoxy resin. These phenomena were also confirmed by the SEM analysis. The TGA and PCFC measurements showed the decrease in the thermal stability of the epoxy resins containing the pure phenolic components, and it was mainly attributed to the early decomposition of the phenolic compounds. Nevertheless, this decrease in thermal stability was accompanied by an increase in char yield and reduction of flammability. The THR was reduced by 10% and the pHRR by 45% with 10 wt.% of gallic acid compared to that of the thermoset without FR. The char content of the epoxy resin was doubled with the incorporation of 10 wt.% of ellagic acid. It could be explained by the interaction of EA with the epoxy matrix as observed in the DSC analysis. The derivatives of gallic and ellagic acids also showed the improvement of thermal characteristics. The pHRR and the THR values were reduced by about 10% by using of 10 wt.% of the phenolic derivatives. The combination of both PCFC and cone calorimeter analyses has highlighted that the significant improvement of flame retardant properties was related to a physical barrier effect. These results prove the potential of the bio-sourced phenolic compounds such as gallic and ellagic acid, as well as their derivatives, as FRs for epoxy resin.

The development of bio-based FRs is still the main purpose of the FR field. In view of the obtained results, the efficiency of the plant extracts (for example, chestnut wood extract) as FR agents for a thermoset matrix should be investigated. The efficiency of its functionalized forms is also proposed to be studied in following works. It is also suggested to investigate the influence of EA on the cure kinetic of the epoxy thermoset by the other methods. The uniform dispersion of the additives in the epoxy matrix should be achieved in order to better evaluate the influence of the phenolic additives on the fire behavior of the epoxy thermosets.

## Figures and Tables

**Table 1:** MS, ^11^B NMR and inductively-coupled plasma–atomic emission spectrometer (ICP–AES) results of the flame retardant (FR) agents.

Sample	Molecular Weight (g mol^−1^)	Main MS Signal (m/z)	^11^B Shifts (ppm)	B_Th_^1^ (wt.%)	B_ICP_^2^ (wt.%)	Na_Th_^3^ (wt.%)	Na_ICP_^4^ (wt.%)
GA	170	125, 169	-	-	-	-	-
EA	302	301	-	-	-	-	-
GAD	-	241, 263	7.7, 13.9	4.23	3.69 ± 0.03	5.37	4.91 ± 0.02
EAD	-	373, 413, 643	1.9, 8.1, 13.1	4.35	3.51 ± 0.02	9.09	7.74 ± 0.03

^1^ B_Th_—theoretical boron content; ^2^ B_ICP_—boron content measured by ICP–AES analysis; ^3^ Na_Th_-theoretical sodium content; ^4^ Na_ICP_ —sodium content measured by ICP–AES analysis.

**Table 2:** Thermal data of the FR agents obtained from thermogravimetric analysis (TGA).

Sample Name	T_max1_ (°C)	T_max2_ (°C)	T_max3_ (°C)	Char_600_ (wt.%)	Char_900_ (wt.%)
GA	269	327	-	25.7	19.3
EA	496	528	630	52.9	31.7
GAD	528	-	-	60.7	53.1
EAD	628	-	-	83.5	52.7

**Table 3:** Data from PCFC tests of the FR systems.

FR systems	pHRR (W g^−1^)	T_peak_ (°C)	THR (kJ g^−1^)	EHC_theo_ (kJ g^−1^)	EHC_exp_ (kJ g^−1^)
GA	293	276	12.4	14.8	15.9
EA	73	492	2.9	16.0	4.5
GAD	10	532	0.3	-	0.6
EAD	25	648	0.5	-	1.0

**Table 4:** Data from DSC analyses of the thermosets.

Thermoset	T_p_ (°C)	$\Delta H\;(J\;g^{-1})$	% Reduction	$\Delta H\;(\mathrm{kJ}\;{\mathrm{ee}}^{-1})$	T_g_ (°C)
DGEBA/IPDA	109	431	-	96	144
DGEBA/IPDA/GA	107	382	11	94	127
DGEBA/IPDA/EA	99	356	17	88	137
DGEBA/IPDA/GAD	105	390	9	95	108
DGEBA/IPDA/EAD	108	391	9	96	110

**Table 5:** Data from TGA analyses of the FR/epoxy systems

Thermoset	Theoretical	Experimental		
	Char_900_^th^	T_max_ (°C)	Char_600_ (wt.%)	Char_900_ (wt.%)
DGEBA/IPDA	-	370	11.3	9.8
DGEBA/IPDA/GA	10.7	349	12.6	10.9
DGEBA/IPDA/EA	12.0	338	20.7	18.6
DGEBA/IPDA/GAD	14.1	367	18.0	15.6
DGEBA/IPDA/EAD	14.1	371	18.0	15.8

**Table 6:** Data from the PCFC tests of the formulated epoxy thermosets.

Thermoset	pHRR (W g^−1^)	% Reduction	T_peak_ (°C)	THR (kJ g^−1^)	EHC_exp_ (kJ g^−1^)
DGEBA/IPDA	525	-	370	28.4	31.8
DGEBA/IPDA/GA	292	45	355	25.8	29.3
DGEBA/IPDA/EA	471	10	343	25.9	32.3
DGEBA/IPDA/GAD	451	14	377	25.3	30.4
DGEBA/IPDA/EAD	463	12	373	27.4	29.7

**Table 7:** Data from the cone calorimeter (heat flux 35 kW m^−2^) test.

Thermoset	TTI (s)	pHRR (kW m^−2^)	% Reduction	THR (kJ g^−1^)	Char (wt.%)
DGEBA/IPDA	90	1116	-	27.0	3.9
DGEBA/IPDA/GA	66	984	12	23.8	8.8
DGEBA/IPDA/EA	80	620	44	23.4	11.2
DGEBA/IPDA/EA*^1^	55	492	56	23.7	12.5
DGEBA/IPDA/GAD	69	394	65	23.8	13.7
DGEBA/IPDA/EAD	75	769	31	25.5	10.0

^1^ The sample DGEBA/IPDA/EA* was exposed to ignition by the side with higher EA content

**Table 8:** ICP–AES results of the char formed during the combustion of DGEBA/IPDA/GAD and DGEBA/IPDA/EAD.

Sample Residue	B_Th_^1^ (wt.%)	B_ICP_^2^ (wt.%)	Na_Th_^3^ (wt.%)	Na_ICP_^4^ (wt.%)
DGEBA/IPDA/GAD	3.84	3.05 ± 0.02	4.26	4.21 ± 0.05
DGEBA/IPDA/EAD	5.39	4.14 ± 0.20	6.84	9.42 ± 0.03

**Table 9:** Boration data of the phenolic compounds.

Sample Name	Phenolic Compound	Ph (mmol)	BA (mmol)	SH (mmol)
GAD	GA	1.7	1.7	1.0
EAD	EA	0.5	1.0	1.0

**Table 10:** Composition of the formulated epoxy thermosets.

Resin name	DGEBA (wt.%)	IPDA (wt.%)	FR (wt.%)
DGEBA/IPDA	80	20	0
DGEBA/IPDA/GA	72	18	10
DGEBA/IPDA/EA			
DGEBA/IPDA/GAD			
DGEBA/IPDA/EAD			

## Supplementary Materials

The calculation of B_Th_ and Na_Th_ contents of GAD and EAD are available online.
