# Supplementary material for: New Biosourced Flame Retardant Agents Based on Gallic and Ellagic Acids for Epoxy Resins
Source: Molecules. 2019 Nov 26;24(23):4305. doi: 10.3390/molecules24234305 (PMC6930652; doi:10.3390/molecules24234305)
Supplement: Supplementary file 1 [file molecules-24-04305-s001.pdf]

New biosourced flame retardant agents based on gallic and ellagic acids for epoxy resins

Valeriia Karaseva [1,2,3], Anne Bergeret [2], Clément Lacoste [2], Hélène Fulcrand [4] and Laurent Ferry [2,*]

[1] INRA, UMR 1083 SPO, 2 place Pierre Viala, 34060 Montpellier, France; valeriia.karaseva@supagro.inra.fr (V.K)
[2] Centre des Matériaux des Mines d'Alès (C2MA), IMT Mines Alès, Université de Montpellier, 6 avenue de Clavières, 30319 Alès cedex, France; anne.bergeret@mines-ales.fr (A.B); clement.lacoste@mines-ales.fr (C.L.)
[3] French Environment and Energy Management Agency, 20 avenue du Grésillé, BP 90406, 49004 Angers cedex 01, France.
[4] Montpellier-SupAgro, UMR 1208 IATE, 2 place Pierre Viala, 34060 Montpellier, France; helene.fulcrand@inra.fr (H.F.)
* Correspondence: laurent.ferry@mines-ales.fr (L.F.)

Number of pages: 2

Theoretical boron and sodium contents

The theoretical boron (B_{Th}) and sodium (Na_{Th}) content of gallic acid derivatives (GAD) and ellagic acid derivatives (EAD) was estimated according to the following calculation. Taking example of EAD.

1. *Estimation of m (EAD).*

It is worth remaining that EAD were prepared by reaction of EA and boric acid (BA) at pH = 9 in presence of sodium hydroxide (SH). EAD was then recovered by freeze-drying of the reaction solution. In such a case, the mass of recovered EAD should be equal the sum of the mass of the solid products used (EA, BA and SH).

The mass of each solid product used can be calculated by multiplying its molar weight by its amount (given in Table 9 of the manuscript). Thus,

$$m\ (EA) = 302.19 \times 0.0005 = 0.151\ g;$$

$$m\ (BA) = 61.83 \times 0.001 = 0.062\ g;$$

$$m\ (SH) = 39.99 \times 0.001 = 0.040\ g;$$

$$m\ (EAD) = m\ (EA) + m\ (BA) + m\ (SH) = 0.253\ g.$$

2. *Estimation of m (B) et m (Na) in EAD.*

The mass of boron (B) and sodium (Na) in EAD can be then calculated respectively by multiplying the proportion of element in BA and SH by the mass of BA and SH in EAD (calculated above). Thus,

$$m\ (B)\ \text{in EAD} = 0.1748 \times 0.062 = 0.011\ g;$$

$$m\ (Na)\ \text{in EAD} = 0.5748 \times 0.040 = 0.023\ g.$$

3. *Estimation of B_{Th} et Na_{Th} of EAD.*

The theoretical boron (B_{Th}) and sodium (Na_{Th}) contents of EAD can be finally calculated as a ratio of the mass of element in EAD (calculated above) to the mass of EAD (calculated above) respectively. To express the content in wt. %, the obtained values can be multiplied by 100. Thus,

$$B_{Th} = 0.011 \div 0.253 \times 100 = 4.35\ \text{wt. \%};$$

$$Na_{Th} = 0.023 \div 0.253 \times 100 = 9.09\ \text{wt. \%}.$$
